# Current News and Opinion

**Published:** 1887-11

**Authors:** 


### Current News and Opinion.

### DENTAL SQUIBBS.

BY "MEANDER." BROOKLYN, N. Y.

"The spirit of the age is essentially practical, and it has little patience with abstract reasoning. We want what can be demonstrated. *Quod erat demonstrandum* is the summing up of all." So says the editor of one of our best dental journals. We sit in silent worship and cry, Amen!

"*What can be demonstrated,*" is the correct text.

Can mortal man "demonstrate" that white is black, and not white at all? Doctor Black says in this Journal, on page 465, Vol. VIII., that "The plastic exudate thrown out in the process of inflammation forms the matrix in which the ameboid cells develop. These cells are always found imbedded within this exudate, and it is"—that is, this exudate—"essential to their"—'their' refers to the ameboid cells—"growth or development into tissue. In the *formation of pus*, this plastic exudate is observed to *liquefy*." Remember this point, "*liquefy!*" "The ameboid cells are found floating in the liquid mass, and are then known as pus corpuscles. Indeed, the cells under these circumstances are no longer capable of development. The matrix in which they are lodged and in which, normally, their development *should* proceed, *has become liquid*, and is *so changed in its chemical qualities that it fails to support them*, and they"—that is, the ameboid cells—*die*.

The italics are the writer's.

The "plastic exudate" is the "matrix." The "plastic exudate" in inflammations *liquefies*, and liquefying causes the death of the ameboid cells. This is the only conclusion of the doctor's argument, for he says so!

Now, then, on page 466, he says: "And the liquefaction of this exudate, carrying with it the ameboid cells in the form of pus, is also constantly proceeding." "If the former"—that is, the liquefaction of this exudate—"exceeds the latter"—that is, the ameboid cells—"the healing of the wound is being accomplished." Above, he tells us where this exudate becomes liquefied it kills these ameboid cells. Then how on earth can the liquefying of this exudate which *kills* the "ameboid cells that come to the surface, tend to pile up in form of living granulations?"

Can such contra-conditions, the one absolutely opposed to the other, be present at one and the same time, *i. e.*, life and death at the same time? Nay, verily!

Will such teachings as these develop in the earnest student lofty attainments in physiology and histology? Again, nay, verily!

Would it not be wise, indeed, is it not absolutely necessary for our several college faculties *to know*, beyond peradventure, that their candidates for graduation can perform the most simple operations on the teeth, with hard or soft gold, in such a manner that said operations will remain as placed, for a period longer than three weeks? Ought not all diplomas to tend to elevate, on a critical analysis, the profession which it represents, rather than to bring discredit, first to the names thereunto attached, and secondly, to the college from which such inefficient persons were professionally born?

Who is the first man to arise and say that he has seen a tooth having a live nerve in itself, and an abscess on its root, at one and the same time? Some wiseacre, who has seen everything, may say: "Humpty-dumpty, why! I've seen such a condition a half dozen times or more." Well, if he has, we never have but once. But we have a case now on hand, and it has beaten our record so completely that we have taken special delight in running a fine probe up, just within the pulp chamber, and then *very innocently* inquiring, "why! did that hurt you so?" Even drawing, sucking on the tooth hurts it severely, and at the same time we have cut into an abscess on the labial surface and evacuated a large amount of pus. The tooth is not sore, does not ache, though nothing is in the cavity but a pledget of cotton wet in carbolic acid and cloves. If we have said it once, we have said it thousands of times, "A tooth cannot ulcerate if you will keep the nerve alive." We believed it, and believe it now as a whole, but these two seemingly opposing and conflicting conditions are just as we have related them. When will fools die and wonders cease? Microbes and bugs can't help us much here.

There are many cases where a cavity has decayed so low, or high, as to take in a considerable portion of the cervical wall—that is, go below the gum. It is well known that oxy-chloride and oxy-phosphate of zinc will too easily dissolve away at the borders of the cervical wall, and a hole burrow underneath the mass of such fillings, and go post-haste for the nerve. To prevent this, use gutta-percha at cervical border, extending up the edges of the cavity at least one-third the approximate wall. One can make only a light gutta-percha approximate wall, and fill in back and behind this with cements. The gutta-percha won't be affected by those fluids that dissolve out the cements, though not hard enough to wear in the more superior attrition surfaces.

---

"Meander" should amend his nomenclature, or he, in turn, may become the subject of criticism. He says "nerve" when he means "pulp" and "ulcer" when evidently there was but a simple abscess. His criticisms upon the paper of Dr. Black are rather hypercritical, and outside the meaning intended by the author.

As regards the "ulcer" connected with the live "nerve," is he certain that the disturbance was not connected with the dead pulp of another tooth? We have seen such appearances when careful search found the origin of the abscess some distance away, and having no connection with the tooth-root over which the fistula opened. An abscess may arise from other causes than a dead pulp, but a live and healthy pulp will not be connected therewith.—EDITOR.

---

### DINNER TO PROF. BUSCH.

A reception and dinner, complimentary to Prof. Busch, of the University of Berlin, was given by Dr. C. F. W. Bödecker on the evening of Sept. 30th, at the residence of the latter, No. 60 East 58th Street, New York.

The gentlemen present were Drs. W. H. Atkinson, Frank Abbott, C. F. W. Bödecker, Prof. Busch, Drs. W. Carr, W. H. Dwinelle, C. E. Francis, Carl Heitzmann, S. G. Perry, C. A. Timme, and W. J. Younger, of San Francisco.

A most excellent dinner, liberally garnished with a variety of choice "extras," gave assurance to every one who had the good fortune to test its merits that the big-hearted and generous host was fully equal to the task of entertaining his friends, and in a manner not to be surpassed.

Early in the evening a letter was received from the Editor of the INDEPENDENT PRACTITIONER, which read as follows :

"*My Dear Dr. Bödecker:*

"It is with feelings of genuine regret that I find myself unable to do honor to your guest, Prof. Dr. Busch, and to yourself, by my presence on the happy occasion of your dinner ; but to show you that I am with you in spirit, if absent in body, I beg to send the following sentiment :

"The Dentists of Germany and America—leaders in theory and in practice. Without the former, the latter is empiricism ; without the latter, the former is pedantry. The German-American should unite the two and make the perfect dentist. Very truly yours,

"W. C. BARRETT."

The sentiment was duly honored, and the health of Dr. Barrett was also "treated" by a rising vote of the company.

Words sparkling with wit and freighted with wisdom were freely and fitly spoken. Scientific and medical subjects bearing upon our specialty were discussed in a conversational way, toasts were offered and each sentiment received a suitable response, making the occasion a feast for both mind and body. Even our worthy and nimble-tongued "patriarch," without whose presence such a gathering would be incomplete, outdid himself in generous utterance of rich aphorisms, which were heartily appreciated by his hearers.

After dinner Prof. Busch was escorted by his friends to the steamship Elbe, (lying at a Hoboken pier), where he was met by a delegation of German students from the New York College of Dentistry, who had assembled to pay their respects to the distinguished visitor, to wish him a safe and pleasant voyage, and to bid him a kindly farewell.

The steamer left for Bremen on the following morning at 5 o'clock.

Dr. Busch is superintendent of the dental departments of the Universities throughout all Germany.

C. E. F.

---

On Wednesday, Sept. 28, Messrs. A. Muller, L. Hern, G. Gudeville and A. Bandman entertained Prof. Busch at a dinner at Martinelli's, Fifth Avenue, New York City, in behalf of the German students of the New York College of Dentistry. The entertainment was a very pleasant one to both guest and hosts.

B.

### NEW ENGLAND DENTAL SOCIETY.

The twenty-fifth annual meeting of this Society was a success from whatever standpoint it may be viewed. It was held in Boston, Oct. 5th, 6th and 7th, and the members in attendance and the character of the papers, discussions and clinics mark a decided era in the history of this old and influential society.

Papers were read by Drs. Gerry, of Lowell; Chupein, of Philadelphia; Shepard, of Boston; Clowes, of New York; Brackett, of Newport; Beers, of Montreal; and Mills, of New York.

Clinical demonstrations were given by Drs. Parr, of New York; Parmly Brown, of Flushing, N. Y.; Carroll, of Meadville, Penn.; Morey, of New York; Waters, of Boston; Freeman, of Chicago; and Genese, of Baltimore.

The officers elected for the ensuing year were:

*President*—Dr. A. M. Dudley, Salem, Mass.

*First Vice-President*—Dr. C. A. Brackett, Newport, R. I.

*Second Vice-President*—Dr. C. W. Clement, Manchester, N. H.

*Secretary*—Dr. A. H. Gilson, Boston, Mass.

*Assistant Secretary*—Dr. W. P. Cooke, Boston, Mass.

*Treasurer*—Dr. G. A. Gerry, Lowell, Mass.

*Librarian*—Dr. E. O. Kinsman, Cambridge, Mass.

*Executive Committee*—Dr. R. R. Andrews, Cambridge, Mass.; Dr. H. A. Baker, Boston, Mass.; Dr. Geo. C. Ainsworth, Boston, Mass.; Dr. W. E. Page, Boston, Mass.; Dr. T. W. Clements, Brookline, Mass.

---

### CONNECTICUT VALLEY DENTAL SOCIETY.

The twenty-fourth annual meeting of this Society will be held at the Hotel Warwick, Springfield, Mass., Thursday and Friday, October 27 and 28, 1887. Papers will be read as follows:

1. Reports from the Ninth International Medical Congress. Dr. L. D. Shepard, Dr. James McManus.
2. The Relation of Medicine to Dentistry. Dr. C. A. Brackett.
3. A Consideration of Methods of Saving Badly Decayed Teeth. Dr. C. T. Stockwell.
4. Retrospect: or Some Reflections based upon the Experience and Observations of Fifty Years of Dental Practice. Dr. F. Searle.
5. Professional Brotherhood. Dr. H. C. Merriam.
6. Teeth with Exposed Pulps. Dr. W. O. Barrett.

All practicing dentists are invited to attend.

GEO. A. MAXFIELD, D. D. S., Secretary.

---

### THE FIRST DISTRICT DENTAL SOCIETY OF THE STATE OF NEW YORK.

Early in the coming January the above Society proposes to hold its Nineteenth Anniversary. To those who have attended previous meetings, under the auspices of the First District, it is hardly necessary to say that it will, in all probability, be a profitable and pleasant gathering. Every opportunity will be offered those who attend to see and hear dentistry from a scientific stand-point. Notwithstanding the great success of some of the previous meetings, the officers propose, if possible, to eclipse all former efforts. Full information will be given next month.

### CORRECTION.

In the report of the International Medical Congress in the October number, page 535, Dr. Younger is said to have recommended the use of *Nitric* acid for necrosed bone. It should read *Lactic* acid.

---

AMONG the foreign representatives to the Dental Section of the recent International Medical Congress was Dr. J. E. Grevers, of Amsterdam, Holland, who was accompanied to this country by his amiable and accomplished wife. The doctor visited many places of interest during his stay, and called on a large number of dentists in New York, Boston, Philadelphia, Buffalo, Chicago, and other cities. He greatly enjoyed his sojourn among us, and seemed delighted with the attention shown him by his friends on this side of the Atlantic.

C. E F.

---

TO-DAY DR. WILLIAM DUTCH, a popular dentist, was found hanging to the transom of his bedroom door. His suicide was due to financial troubles.

—*San Francisco Special, Oct. 24th.*

Dr. Dutch was one of the early graduates of the New York College of Dentistry. He was for one year President of the American Dental Convention, was an excellent operator, and had a large practice in San Francisco.

---

WE KNEW IT MUST COME! We have it from a reliable source, that "Topeka" Thompson is now struggling with a type writer. He has mastered the "Caps," and is now hard at work on the "Lower Case." We venture the assertion that there will be hallelujahs around the office of the *Western Dental Journal* that will loosen the paper on the walls. Now for Harlan, of *The Review.*

---

THE EDITOR of this Journal will pay a liberal price in cash for the following numbers of dental journals, or he will exchange other numbers for them:

THE DENTAL REGISTER—Vol. III, Nos. 1, 2, 3. Vol. VI, No. 1.

THE AMERICAN JOURNAL OF DENTAL SCIENCE—Third Series—Vol. VII, Nos. 7, 10. Vol. VIII, No. 7.

---

THE MANY PROFESSIONAL FRIENDS of Dr. E. D. Downs, of Owego, will learn with great regret that he is now in New York City for the purpose of having his leg amputated at the thigh, for a disease caused by an injury received from the crank of his dental chair.

---

THE CLERGY and the Jews were the leading men of the medical profession during the tenth and eleventh centuries. From 1131 down to 1161 the Popes took occasion to thunder against practicing ecclesiastics.

---

A TRAVELING DOCTOR who is holding forth in Indiana, has his bills read: If not hung by a mob, I shall reach this place about———.

---

HENRY V. at Agencourt, with 30,000 men, had one surgeon and fifteen assistants.

