# Correction

**Published:** 1888-02

**Authors:** 

**Affiliations:** Director of the Dental Institute


### CORRECTION.

*Editor Independent Practitioner:*

In the November number of your excellent Journal is found the following sentence : "Dr. Busch is Superintendent of the Dental Department of the Universities throughout Germany." This is a mistake which, if not corrected, may be the source of much inconvenience to me, and I therefore take the liberty to ask for a correction as follows : Dr. Busch is Professor Extraordinary in the Medical Faculty of the University of Berlin, and Director (superintendent) of the Dental Department. PROF. DR. BUSCH,

Director of the Dental Institute.

